# Factors Associated With the Risk of Progression of Low-Risk Branch-Duct Intraductal Papillary Mucinous Neoplasms

**DOI:** 10.1001/jamanetworkopen.2020.22933

**Published:** 2020-11-30

**Authors:** Gabriele Capurso, Stefano Crippa, Giuseppe Vanella, Mariaemilia Traini, Giulia Zerboni, Piera Zaccari, Giulio Belfiori, Manuel Gentiluomo, Tommaso Pessarelli, Maria Chiara Petrone, Daniele Campa, Massimo Falconi, Paolo Giorgio Arcidiacono

**Affiliations:** 1 Pancreato-biliary Endoscopy and Endoscopic Ultrasound, Pancreas Translational and Clinical Research Center, San Raffaele Scientific Institute IRCCS, Milan, Italy; 2 Digestive and Liver Disease Unit, S Andrea Hospital, Rome, Italy; 3 Pancreatic Surgery Division, Pancreas Translational and Clinical Research Center, San Raffaele Scientific Institute IRCCS, Milan, Italy; 4 Università Vita-Salute, Milan, Italy; 5 Department of Biology, University of Pisa, Pisa, Italy

## Abstract

**Importance:**

Branch-duct intraductal papillary mucinous neoplasms (BD-IPMNs) are common pancreatic preneoplastic lesions, but their surveillance is not personalized.

**Objective:**

To investigate patient- and cyst-related factors associated with progression into worrisome features (WFs) or high-risk stigmata (HRS) categories of BD-IPMNs.

**Design, Setting, and Participants:**

Cyst- and patient-related factors of consecutive BD-IPMNs without WFs or HRS in 540 patients diagnosed from 2009 to 2018 with at least 12 months’ surveillance until February 28, 2020, were registered in a 2-center ambispective cohort study in Italy. In a subgroup, the ABO blood group was studied for the first time in this setting.

**Exposure:**

Cyst-related and patients-related factors and ABO blood group.

**Main Outcomes and Measures:**

The study outcome was the appearance of WFs or HRS according to the 2017 International Association of Pancreatology guidelines. Survival probability was calculated using Kaplan-Meier curve and risk factors identified by Cox proportional hazards regression. ABO blood group was inferred through genotypes with DNA extraction.

**Results:**

Of 540 patients with BD-IPMNs (median age, 66 years [interquartile range, 58.5-72.0 years]; 337 women [62.4%]) undergoing surveillance for a median of 51.5 months (interquartile range, 28-84 months) for 2758 person-years, 130 patients (24.1%) experienced progression. Probability of progression was 3.7% at 1 year, 23.4% at 5 years, and 43.3% at 10 years; 15 patients (2.8%) underwent surgery, 7 patients (1.3%) had malignant histologic findings, and 3 patients (0.56%) died of pancreatic-associated disease. Initial cyst size greater than 15 mm (hazard ratio [HR], 2.05; 95% CI, 1.44-2.91), body mass index greater than 26.4 (HR, 1.72; 95% CI, 1.19-2.50), and heavy smoking (HR, 1.81; 95% CI, 1.14-2.86) were significant independent factors associated with progression risk. The AA blood genotype was also associated with progression risk (HR, 3.49; 95% CI, 1.04-11.71) compared with the OO genotype in the investigated subgroup.

**Conclusions and Relevance:**

This analysis of factors associated with progression of BD-IPMNs according to recent guidelines suggests that cyst size alone is not a reliable factor for estimation of progression risk; however, along with other readily available data, size is helpful for planning personalized surveillance of BD-IPMNs.

## Key Points

**Question:**

Can factors that are readily available at diagnosis be used to estimate the risk of progression of branch-duct intraductal papillary mucinous neoplasms (BD-IPMNs)?

**Findings:**

In this cohort study that included 540 patients under surveillance for a median of 51.5 months, initial cyst size greater than 15 mm, body mass index greater than 26.4, and heavy smoking were associated with progression risk of BD-IPMNs. The AA blood genotype was also associated with progression risk.

**Meaning:**

Results of this study suggest that cyst size alone is not a reliable factor to estimate progression risk, but along with other readily available data, size is helpful for planning personalized surveillance of BD-IPMNs.

## Teaser

This cohort study examines the use of patient- and cyst-related factors available at baseline for estimation of the risk of progression in patients with branch-duct intraductal papillary mucinous neoplasms.

## Introduction

Intraductal papillary mucinous neoplasms (IPMNs) are common incidental findings^1^ and considered preneoplastic lesions that could progress to pancreatic ductal adenocarcinoma (PDAC).^2,3^

When IPMNs harbor morphologic features associated with cancer risk, surgery is indicated, and surveillance is advised for most patients with good life expectancy.^2^ Guidelines for IPMN management underline the importance of specific morphologic features associated with increased risk of cancer,^4,5,6^ including size of the branch-duct (BD) cyst, presence of solid nodules, and main pancreatic duct dilation. Most IPMNs, however, are small BD lesions without such features and carry a low risk of cancer.^3^

There have been efforts to investigate characteristics associated with the subsequent progression risk. The initial size and multifocality of BD-IPMNs have been associated with progression risk during surveillance.^7,8,9,10^ These results, however, were based on older guidelines and did not consider IPMNs’ growth rate.^11,12^ Also, information on possible patient-related factors associated with progression risk is limited.^9,10^

In addition to the limited knowledge of cyst and patient features, there are few available data on the role of genetic variants in this context. Because it has been reported that non–type O blood is associated with an increased risk of PDAC,^13,14^ we hypothesized that this variant might also be associated with the risk of progression of BD-IPMN.

The present study aimed to investigate comprehensively cyst-related and patient-related factors associated with worrisome features (WFs) or high-risk stigmata (HRS) development in a cohort of BD-IPMNs under surveillance. To our knowledge, the role of ABO blood group type was also evaluated in a subgroup for the first time in this setting.

## Methods

### Study Design

This 2-center, ambispective (retrospective and prospective) cohort study was conducted in tertiary centers in Italy (S Raffaele Research Hospital, Milan, and Sant’Andrea Hospital, Rome), following the Strengthening the Reporting of Observational Studies in Epidemiology (STROBE) reporting guideline. All consecutive patients with suspected BD-IPMNs diagnosed between 2009 and 2018, were screened for inclusion with follow-up until February 28, 2020, being recorded. Prospective data collection began after institutional review board approval by Sant’Andrea Hospital and S Raffaele Research Hospital. Additional institutional review board approval was obtained for consecutive patients in whom blood group was investigated. Written informed consent was obtained for both study phases. Patients did not receive financial compensation, and data were deidentified.

Inclusion criteria were (1) BD-IPMN diagnosis with typical findings observed on magnetic resonance imaging with magnetic resonance cholangiopancreatography and/or on endoscopic ultrasonography (ie, presence of ≥1 dilated BD [≥5 mm]) communicating with a nondilated main pancreatic duct (<5 mm),^15,16^ (2) follow-up of 12 months or longer, and (3) age greater than 18 years.

Exclusion criteria were the presence of WFs and HRS at diagnosis according to the International Association of Pancreatology guidelines^11^ and/or atypical cytologic factors (high-grade dysplasia or cancer). Worrisome features were defined as (1) cyst size 3 cm or larger, (2) enhancing mural nodule less than 5 mm, (3) thickened enhanced cyst walls, (4) main pancreatic duct size between 5 and 10 mm, (5) abrupt change in main pancreatic duct diameter with distal pancreatic atrophy, (6) lymphadenopathy, (7) increased serum cancer antigen 19-9 level, (8) cyst growth rate greater than 5 mm within 2 years, and (9) acute pancreatitis. High-risk stigmata were defined as (1) IPMNs causing obstructive jaundice, (2) enhancing mural nodules greater than or equal to 5 mm, and (3) main pancreatic duct greater than or equal to 10 mm. Patients with a cyst less than 5 mm, chronic pancreatitis or pancreatitis diagnosed during or immediately after acute pancreatitis, concomitant or previous diagnosis of solid pancreatic tumor, or previous pancreatic surgery were also excluded. At diagnosis, trained physicians interviewed patients on demographic characteristics, medical history, risk factors, and symptoms.

Patients underwent annual or semiannual surveillance with contrast-enhanced magnetic resonance imaging and magnetic resonance cholangiopancreatography according to the International Association of Pancreatology guidelines.^11^ Endoscopic ultrasonography with or without cystic fluid aspiration and/or fine-needle aspiration was performed when progression and/or appearance of WFs or HRS occurred. After initial magnetic resonance imaging, the maximum cyst diameter was measured in the same scan plane of the index examination. During surveillance, patients were seen in dedicated outpatient clinics.

### Study Outcomes and Variables

During surveillance, clinicoradiologic changes compared with baseline characteristics were analyzed. The study end point was the progression to the WFs or HRS category, whichever occurred first. The agreement of 2 staff members (G.C. and S.C.) was required to define progression. Data on the need for surgery and pathologic findings in patients who underwent resection and on overall and disease-specific survival were also recorded.

Patient-related factors at time of diagnosis that were considered possible explanatory variables included age, sex, body mass index (BMI) (calculated as weight in kilograms divided by height in meters squared), previous diabetes diagnosis and insulin use, smoking, alcohol intake, pancreatic cancer and any other cancer, and family history of pancreatic or any other cancer. Possible cyst-related factors included the diameter of the largest cyst and the number of cysts (unifocal or multifocal). In multifocal BD-IPMNs, the largest lesion diameter was recorded. In a subgroup of patients, the association between ABO blood group and progression was also investigated. Definitions of exposures were reported in a previous study.^17^ In detail, ever-smokers were defined as individuals reporting more than 6 months of smoking or greater than 100 cigarettes smoked during their lifetime. The total amount of smoking was evaluated as pack-years, defined as packs smoked per day × total years of smoking, with 20 pack-years set as the cutoff to define heavy smokers. Ever-alcohol drinkers were individuals drinking a mean of 12.5 g or more of alcohol per day for at least 1 year or a lower amount for more than 1 year. One glass of wine, 1 pint (or can) of beer, and 1 shot of hard liquor were considered equal to 1 alcohol unit (approximately 12.5 g of alcohol). A cutoff of 21 U/wk (262.5 g) was defined as heavy drinking. Overweight was defined as BMI greater than or equal to 25 and obesity as BMI greater than or equal to 30.

During surveillance, patients developing WFs and/or HRS were evaluated for surgical resection at multidisciplinary boards as appropriate. If patients died during surveillance, the date and cause of death were recorded. For the study purposes, IPMN-distinct PDAC and IPMN with high-grade dysplasia or invasive carcinoma were considered as malignant tumors and causes of pancreas-related death.

### ABO Blood Group Evaluation

The ABO blood group was inferred through genotypes with DNA extraction (QIAmp DNA Mini Kit or AllPrep isolation kit, Qiagen). Genotyping was conducted using a polymerase chain reaction–based genotyping system (KASPar SNP, KBiosciences). Genotypes calls were done using real-time polymerase chain reaction (ViiA 7 Real-Time PCR System, ThermoFisher Applied Biosystems). For blood group computation, we used rs505922 to distinguish between O and non-O, and rs8176746 from ABO A and B alleles. Using the genotyping combination of these 2 single-nucleotide variants, the blood group was reconstructed.^18^

### Statistical Analysis

Continuous variables are presented as mean (SD) when distribution is normal or as median (25th-75th interquartile range [IQR]) when skewed. Length of surveillance was measured (expressed in months) from the initial IPMN diagnosis to (1) last surveillance examination for patients who remained alive, (2) date of death for patients who underwent resection, and (3) date of surgery for patients who underwent resection. Progression-free survival was defined as the time between diagnosis and progression to either WFs or HRS. Survival analysis was performed using the Kaplan-Meier estimator, with results compared using a log-rank test. The association between investigated variables and end points is expressed as hazard ratio (HR) (95% CI). A receiver operating characteristic (ROC) curve was constructed to determine the best cutoff values of BMI and cyst size associated with progression, using the Youden index J.

The analysis of risk factors associated with progression-free survival was performed with univariable and multivariable analysis using the Cox proportional hazards regression model, since the association with the investigated variables was assumed to be constant over time. The multivariable model was constructed by the enter method. The enter method forces the inclusion of selected independent variables in the model and is preferred when the selected variables are all perceived to be relevant in predicting the event. The selected variables were those significant at the univariable analysis. A 2-sided *P* < .05 was considered statistically significant. A dedicated software program (Medcalc, version 12.1) was used.

## Results

### Study Population

With a total of 747 patients screened during the study period, a cohort of 540 patients (72.3%) was included in the analysis. Of these, 337 patients (62.4%) were women; median age was 66 years (IQR, 58.5-72.0 years). One hundred thirty-nine patients were excluded because they had at least 1 WF or HRS at diagnosis, 29 were excluded because the cyst was less than 5 mm or IPMN diagnosis did not meet criteria for suspected at imaging revision, 11 patients refused to give informed consent, and 28 patients had a follow-up visit at less than 12 months or missed planned surveillance. The cohort characteristics are summarized in Table 1. Of these 540 patients, the ABO group was determined in 89 consecutive cases enrolled after approval of the biobanking study.

**Table 1.** Cyst- and Patient-Related Factors at Diagnosis

Characteristic	No. (%)
Patient-related factors	
No.	540
Female sex	337 (62.4)
Age at diagnosis, median (IQR), y	66 (58.5-72.0)
BMI, median (IQR)	24.5 (22.7-26.7)
Diabetes	75 (13.9)
Insulin use	13 (2.4)
Ever smoker	192 (35.5)
Heavy smoker (>20 pack-years)	64 (11.9)^a^
Alcohol drinker	166 (30.7)
Heavy alcohol drinker (>21 U/wk)	21 (3.9)
1st-degree family history of any cancer	152 (28.1)
1st-degree family history of pancreatic cancer	27 (5.0)
Personal history of previous neoplasia	141 (26.1)
**Cyst-related factors**	
Diameter of largest BD-IPMN, median (IQR), mm	14 (10-20)
Multifocal lesions	289 (53.5)

Abbreviations: BD-IPMN, branch-duct intraductal papillary mucinous neoplasm; BMI, body mass index (calculated as weight in kilograms divided by height in meters squared); IQR, interquartile range.

^a^ Data on amount of smoking missing for 1 smoker.

### Development of WFs or HRS 

During a median surveillance time of 51.5 months (IQR, 28-84 months) for a total of 2758 person-years, a progression toward WFs or HRS was recorded in 130 of 540 patients (24.1%) (Table 2). Of these 130 patients, 127 developed WFs first and only 3 (2.3%) developed HRS without previous WFs. More than 1 WF at the same time appeared in 52 of 127 patients (40.9%). Only 3 patients had 3 WFs, and the remaining 49 patients had 2 WFs develop at the same time; in 43 cases, an increase in size greater than 5 mm occurred within 24 months with a final size greater than 30 mm.

**Table 2.** Outcomes During Surveillance

Outcome	No./No. (%)
Surveillance time, median (IQR), mo	51.5 (28-84)
Progression to either WF or HRS	130/540 (24.1)
Progression to WF as first event	127/540 (23.5)
Size >30 mm	57/127 (44.9)
Increase of size >5 mm within 24 mo	101/127 (79.5)
MPD diameter 5-9 mm	24/127 (18.9)
Enhancing nodule <5 mm	4/127 (3.1)
Acute pancreatitis	3/127 (2.3)
Progression to HRS as first event	3/540 (0.6)
Pancreatic surgery	15/540 (2.8)
High-grade dysplasia or cancer on pathologic testing	7/540 (1.3)
Death from any cause	31/540 (5.7)
Pancreas-related death	3/540 (0.6)

Abbreviations: HRS, high-risk stigmata; IQR, interquartile range; MPD, main pancreatic duct; WF, worrisome feature.

**Figure 1.**
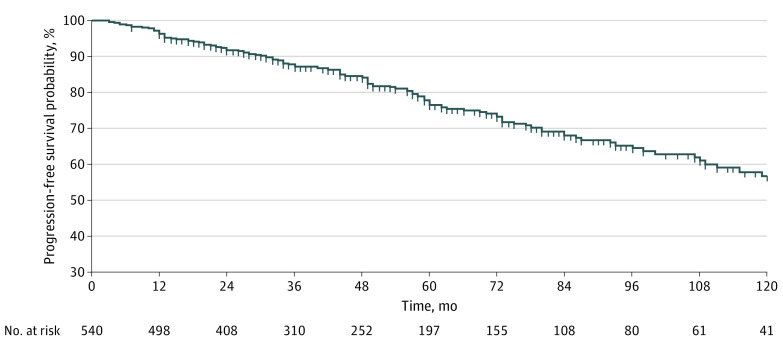
Probability of Survival Free of Progression Into Worrisome Features or High-Risk Stigmata in 540 Patients With Branch-Duct Intraductal Papillary Mucinous Neoplasms Under Surveillance

The median time to development of either WFs or HRS was 45 months (IQR, 25.0-77.5 months). The rate of progression to either a WF or an HRS during surveillance was 47.1 per 1000 person-years (4.7% per year); 39 of 127 events (30.7%) occurred after 5 years of stable findings. Kaplan-Meier curves showed a probability of progression of 3.7% at 1 year, 23.4% at 5 years, and 43.3% at 10 years (Figure 1).

### Surgery, Pancreatic Cancer, and Death 

Fifteen patients (2.8%) underwent surgery after the appearance of HRS (8 patients) or of multiple WFs, with findings of IPMN with high-grade dysplasia in 3 cases, invasive carcinoma in 2 patients, and PDAC arising in distinct parts of the pancreas in 2 patients. The overall cancer rate was 1.3% (7 of 540); the remaining 8 patients had IPMN with mild to moderate dysplasia. No patient who underwent surgery died.

There were 31 deaths (5.7%), but only 3 deaths (0.56%) were pancreas related: 2 occurred owing to recurrence in resected patients with PDAC and 1 in a patient aged 87 years with cancer during surveillance who did not undergo surgery because of age and comorbidities.

### Factors Associated With Progression Risk

An ROC analysis defined a BMI greater than 26.4 (Youden Index J = 0.14) as the best discriminating value associated with progression risk, although with moderate accuracy (area under the ROC, 0.57; sensitivity, 37.7% [95% CI, 29.3%-46.6%]; specificity, 76.6% [95% CI, 72.2%-80.6%]). Regarding the initial cyst size, in the ROC curve analysis, the best dimensional cutoff associated with progression risk was greater than 15 mm (Youden Index J = 0.21) with moderate accuracy (area under the ROC, 0.63; sensitivity, 56.2% [95% CI, 47.2%-64.8%]; specificity, 65.6% [95% CI, 60.8%-70.2%]) (eFigure in the Supplement).

**Figure 2.**
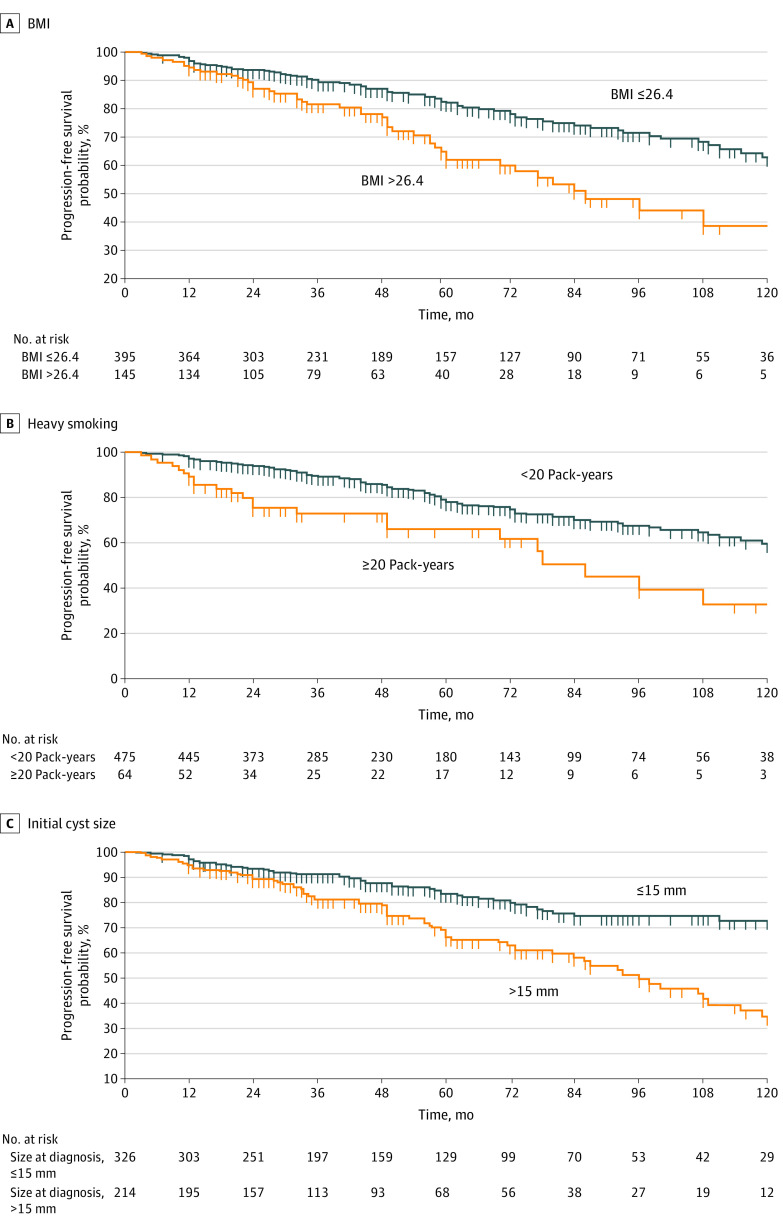
Factors Associated With Probability of Progression Into Worrisome Features or High-Risk Stigmata Factors evaluated included body mass index (BMI) (calculated as weight in kilograms divided by height in meters squared) (A), heavy smoking (B), and initial cyst size greater than 15 mm (C).

As reported in Table 3, in the multivariable analysis, the best fit model was the one including a BMI greater than 26.4 (HR, 1.72; 95% CI, 1.19-2.50; *P* = .004), heavy smoking (HR, 1.81; 95% CI, 1.14-2.86; *P* = .01), and cyst size greater than 15 mm (HR, 2.05; 95% CI, 1.44-2.91; *P* < .001). The Kaplan-Meier curves representing the risk of progression according to the presence or absence of these 3 variables are presented in Figure 2. Although the probability of progression was much lower in patients with an initial cyst size less than or equal to 15 mm, it increased over time with a probability of being progression-free of 90.2% at 3 years, 82.8% at 5 years, and 69.6% at 10 years. We then estimated the progression risk in 4 subgroups: (1) size less than or equal to 15 mm and neither heavy smoking nor BMI greater than 26.4 (reference); (2) size less than or equal to 15 mm, heavy smoking, and BMI greater than 26.4; (3) size greater than 15 mm and neither heavy smoking nor BMI greater than 26.4; and (4) size greater than 15 mm, heavy smoking, and BMI greater than 26.4. The rate of patients free of progression at 5 years was 84.2% in group 1 (reference); lower progression-free rates were noted in all other groups with Cox proportional hazards regression: 57.1% in group 2 (HR, 5.65; 95% CI, 1.75-18.2), 70% in group 3 (HR, 2.22; 95% CI, 1.53-3.22), and 47.9% in group 4 (HR, 4.38; 95% CI, 1.81-10.61) (χ^2^ for comparison among groups, *P* < .001). A history of diabetes, alcohol intake, family or personal history of pancreatic cancer or any other cancer, and the number of cystic lesions were not associated with progression risk.

**Table 3.** Factors Associated With Risk of Progression to Either WF or HRS

Characteristic	Univariable HR (95% CI)	*P* value	Multivariable HR (95% CI)^a^	*P* value
Male sex	1.08 (0.76-1.53)	.64	NA	
Age at diagnosis (per increasing year)	1.08 (0.99-1.02)	.31	NA	
BMI (per increasing unit)	1.07 (1.02-1.12)	.004	NA	
BMI>26.4	2.06 (1.44-2.95)	<.001	1.72 (1.19-2.50)	.004
Diabetes	1.19 (0.73-1.95)	.46	NA	
Insulin-dependent diabetes	0.54 (0.13-2.2)	.54	NA	
Ever smoking	1.57 (1.16-2.21)	.01	NA	
Heavy smoking^a^	2.48 (1.6-3.8)	<.001	1.81 (1.14-2.86)	.01
Alcohol drinker	1.2 (0.83-1.72)	.31	NA	
Heavy alcohol drinker^b^	1.52 (0.71-3.25)	.27	NA	
1st-degree family history of any cancer	1.42 (0.98-1.36)	.06	NA	
1st-degree family history of pancreatic cancer	1.30 (0.66-1.72)	>.99	NA	
Personal history of previous neoplasia	0.84 (0.56-1.25)	.84	NA	
Size of largest BD-IPMN (per increasing mm)	1.06 (1.03-1.09)	<.001	NA	
Size >15 mm	2.27 (1.61-3.21)	<.001	2.05 (1.44-2.91)	<.001
Multifocal lesions	0.94 (0.66-1.23)	.94	NA	

Abbreviations: BD-IPMN, branch-duct intraductal papillary mucinous neoplasm; BMI, body mass index (calculated as weight in kilograms divided by height in meters squared); HR, hazard ratio; HRS, high-risk stigmata; NA, not analyzed; WF, worrisome feature.

^a^ Heavy smoking defined as greater than 20 pack-years.

^b^ Heavy drinking defined as greater than 21 alcohol U/wk.

### Association Between ABO Blood Group and Progression

Genotyping for determination of ABO blood group was performed in 89 consecutive patients with similar characteristics compared with the total cohort (55 women [61.8%]; median age, 65 years (IQR, 59-72 years). The median surveillance time in this subgroup was 74 months (IQR, 46-95 months), and the median cyst size was 15 mm (IQR, 46-95 mm).

We observed no statistically significant association when comparing carriers of the O vs the other blood type groups. However, individuals with the AA genotype had an increased risk of developing WFs or HSR compared with patients with the OO genotype (HR, 4.62; 95% CI, 1.43-15.02; *P* = .01) (eTable in the Supplement). As for other factors, in this subgroup, cyst size greater than 15 mm was a risk factor for progression (HR, 2.4; 95% CI, 1.09-5.33; *P* = .03), and smoking and BMI were not. In a multivariable analysis corrected for cyst size greater than 15 mm, the AA genotype maintained a significant association with the risk of progression (HR, 3.49; 95% CI, 1.04-11.71; *P* = .04).

## Discussion

Most BD-IPMNs have a low risk of malignant transformation. Apart from specific morphologic changes considered overt indicators of the need for invasive diagnostics or operative management, there are few factors guiding clinicians’ choices; thus, follow-up is similar for all patients potentially fit for surgery. This study investigated factors available at diagnosis, including, to our knowledge for the first time in this setting, the ABO blood group that might be associated with the risk of developing WFs or HRS.

In this ambispective cohort of 540 BD-IPMNs under surveillance for a median of 51.5 months, both cyst-related factors, such as initial cyst size, and patient-related factors, such as overweight and heavy smoking, were associated with progression risk. In the subgroup genotyped for ABO group, the AA genotype was associated with increased risk and the non-O group was not.

The observed rate of progression is in keeping with the 3.8% per year, with a mean of 12.8% during a mean follow-up of 3.8 years reported in a meta-analysis^19^ and with the rate of WF development of 14.8% in a mean follow-up of 61 months reported in a cohort of 1310 patients with BD-IPMN.^20^ This rate is lower in studies with shorter follow-up, being 4.2% after 25 months of surveillance among 1036 patients with BD-IPMNs.^21^ The probability of progression was 4% at 1 year, 24% at 5 years, and 43% at 10 years, with some of the events occurring after 5 years of stability. This observation is in keeping with previous studies^16,22,23^ and emphasizes the need to consider with caution^24^ the American Gastroenterology Association 2015 guideline recommendations^5^ to discontinue surveillance of BD-IPMNs after 5 years of no significant changes.

Others have reported the role of initial size in predicting the risk of malignant progression. In a cohort of 577 patients with BD-IPMNs followed up for a median of 82 months,^7^ the same cutoff of 15 mm that we adopted estimated the risk of cancer with a sensitivity of 95% and negative predictive value of 99%. However, that study included 12% of patients who already had WFs at baseline (mostly size >30 mm), possibly enriching the portion of high-risk patients and thus the probability of progression. In our cohort of low-risk BD-IPMNs with a median initial size of 14 mm, the sensitivity and specificity of the 15 mm cutoff to estimate the development of WFs or HRS were 56% and 66%. In the present cohort the progression rate was similar in patients with initial cyst size greater than or less than 15 mm who had a BMI greater than 26.4 and were heavy smokers, suggesting that cyst size alone is insufficient to stratify this risk.

The association between overweight and progression of IPMNs under surveillance is underinvestigated. Obesity has been associated with an increased risk of cancer in operated IPMNs,^25^ and pancreatic fat content with the progression of low-risk BD-IPMNs,^26^ pointing to metabolic factors as possible modulators of IPMN growth.

Smoking is a risk factor associated with the occurrence of IPMNs.^27^ We found that both ever smoking and heavy smoking were associated with progression risk, with, to our knowledge, a previously unreported dose-related association.^9,10^

To our knowledge, this is the first study investigating the association between ABO blood groups and progression risk of BD-IPMNs, although this analysis was performed in just a subgroup. The only previous study investigating blood groups in IPMNs was conducted in a large cohort of surgical patients^28^ in which non-O group was not associated with cancer. As non-O blood group has been consistently associated with an increased risk of developing PDAC,^14,19,20^ our hypothesis was that the non-O blood group might be a risk factor for IPMN progression. However, we observed no statistically significant association. We report a significant association between AA and the risk of progression (adjusted HR, 3.49), although this was not the case for other non-O genotypes. This finding strengthens the hypothesis of the O allele to be protective against PDAC development and confirms a more relevant role of the A group.^13^

### Strengths and Limitations

The present study has strengths, such as the relatively large cohort, the ambispective design, the analysis of a large information set, and the apparent novelty of some findings. Compared with previous studies,^10^ the use of updated guidelines allowed the inclusion of a growth rate more than 5 mm over 24 months among WFs. This result is relevant, as the growth rate of BD-IPMNs has been associated with risk of malignant transformation.^29^ In this view, the European evidence-based guidelines^12^ are similar and their use would have most likely led to comparable results.

The study has limitations. The evaluation of outcomes that are not hard (ie, death or disease that may cause death) is a limitation. However, because the rates of cancer and pancreas-related death are rare in this setting, WFs and HRS are reasonable surrogate outcomes. The possibility of both selection bias because patients were enrolled in tertiary centers and information bias regarding variables’ measure and of residual confounding should be considered. However, all information was collected during face-to-face interviews and was not retrieved by medical records, and imaging findings were carefully evaluated. In addition, given the ambispective design, some information, such as prospective measure of Ca 19-9 levels, is lacking and application of the 2017 International Association of Pancreatology criteria was retrospective in some cases.

The study should be considered exploratory, especially the section on blood groups. In addition, associations for risk factors identified within the same study are likely to be overoptimistic; validation of the present findings is necessary.

## Conclusions

To our knowledge, the findings of this study suggest for the first time that in addition to size, overweight, heavy smoking, and AA blood group are associated with the risk of WFs or HRS development of BD-IPMNs. These variables, readily available at the time of diagnosis, may help in stratifying patients to personalized follow-up strategies.

## Notes

Supplement. **eTable.** Association Between Blood Group and Progression Into Either WF or HRS in a Subgroup of 89 Patients **eFigure.** ROC Curve Investigating Best Cutoff Values Associated With the Risk of Progression Into Worrisome Features or High-Risk Stigmata
