# Supplementary material for: Factors Associated With the Risk of Progression of Low-Risk Branch-Duct Intraductal Papillary Mucinous Neoplasms
Source: JAMA Netw Open. 2020 Nov 30;3(11):e2022933. doi: 10.1001/jamanetworkopen.2020.22933 (PMC7705592; doi:10.1001/jamanetworkopen.2020.22933)
Supplement: Supplement. — eTable. Association Between Blood Group and Progression Into Either WF or HRS in a Subgroup of 89 Patients eFigure. ROC Curve Investigating Best Cutoff Values Associated With the Risk of Progression Into Worrisome Features or High-Risk Stigmata [file jamanetwopen-e2022933-s001.pdf]

Supplementary Online Content

Capurso G, Crippa S, Vanella G, et al. Factors associated with the risk of progression of low-risk branch-duct intraductal papillary mucinous neoplasms. *JAMA Netw Open*. 2020;3(11):e2022933. doi:10.1001/jamanetworkopen.2020.22933

eTable. Association Between Blood Group and Progression Into Either WF or HRS in a Subgroup of 89 Patients

eFigure. ROC Curve Investigating Best Cutoff Values Associated With the Risk of Progression Into Worrisome Features or High-Risk Stigmata

This supplementary material has been provided by the authors to give readers additional information about their work.

© 2020 Capurso G et al. *JAMA Network Open.*

eTable: Association between Blood Group and progression into either WF or HRS in a subgroup of 89 patients.

Variable	No progression (n= 63)	Progression (n= 26)	Univariable HR (95%CI);	p value	Multivariable* HR (95%CI)	p value
**0 group (n= 39)**	27 (69.2%)	12 (30.8%)	1			
**non-0 group (n= 50)**	36 (72%)	14 (28%)	0.87 (0.50-1.53)	0.64	-	
**0 group (n= 39)**	27 (69.2%)	12 (30.8%)	1			
**group A (n= 39)**	26 (66.7%)	13 (33.3%)	1.51 (0.67-3.39)	0.32	-	
**group B (n= 1)**	1 (100%)	0	-		-	
**group AB (n= 10)**	9 (90%)	1 (10%)	0.31 (0.04-2.42)	0.27	-	
**00 genotype (n= 39)**	27 (69.2%)	12 (30.8%)	1		1	
**genotype AA (n= 5)**	1 (20%)	4 (80%)	4.62 (1.43-15.02)	0.01	3.49 (1.04-11.71)	0.04
**genotype A0 (n= 34)**	25 (73.5%)	9 (26.5%)	1.14 (0.47-2.77)	0.77	-	
**genotype AB (n= 10)**	9 (90%)	1 (10%)	0.32 (0.04-2.46)	0.27	-	
**genotype BB (n= 1)**	1 (100%)	0	-		-	

*adjusted for Cyst Size>15 mm. WF= worrisome feature; HRS= high risk stigmata; HR = hazard ratio.

© 2020 Capurso G et al. *JAMA Network Open.*

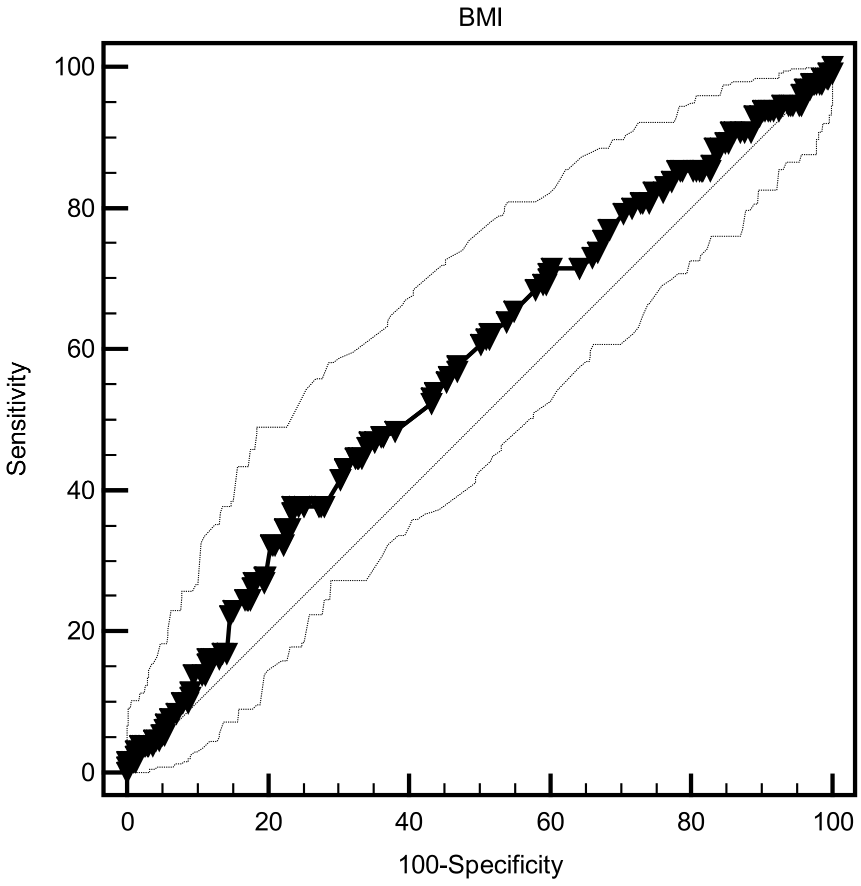

Panel A

eFigure. ROC Curve Investigating Best Cutoff Values Associated With the Risk of Progression Into Worrisome Features or High-Risk Stigmata

The best cutoff value for BMI was 26.4 (panel A) with an area under the curve of 0.57 (sensitivity 37.7%, specificity 71.8%), while the best cutoff for size of the BD cyst was 15 mm (panel B), with an area under the curve of 0.63 (sensitivity 56.2%, specificity 65.6%).

© 2020 Capurso G et al. *JAMA Network Open.*

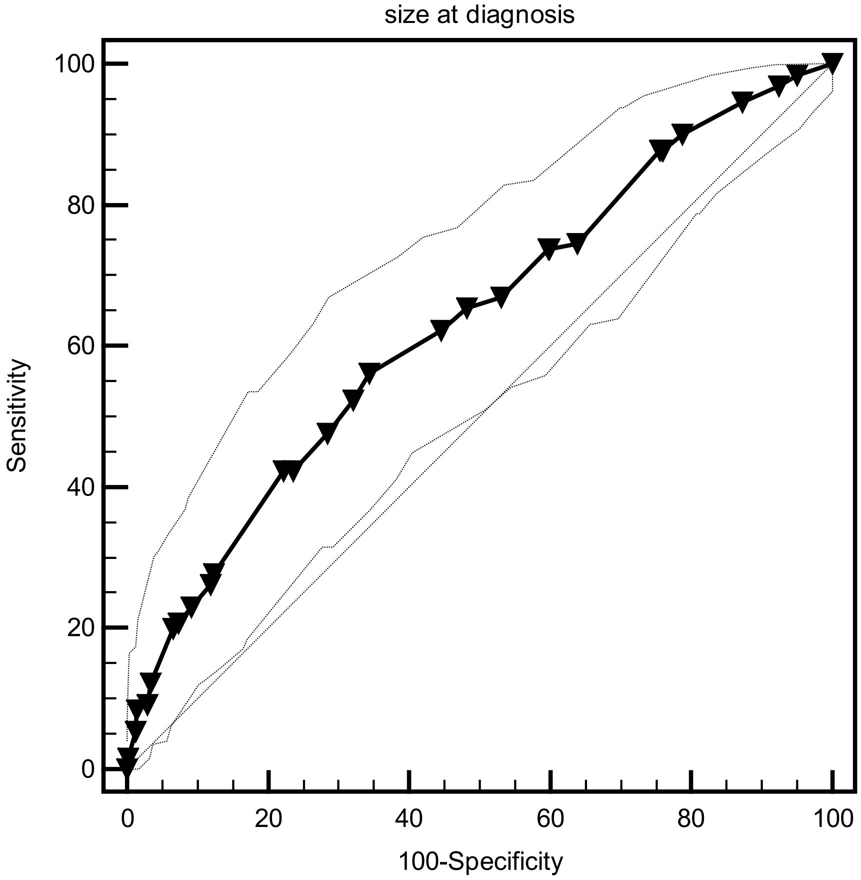

Panel B

© 2020 Capurso G et al. *JAMA Network Open.*
